# Underwater Image Transmission Using Spatial Modulation Unequal Error Protection for Internet of Underwater Things

**DOI:** 10.3390/s19235271

**Published:** 2019-11-29

**Authors:** Hamada Esmaiel, Zeyad A. H. Qasem, Haixin Sun, Junfeng Wang, Naveed Ur Rehman Junejo

**Affiliations:** 1 Department of Information and Communication, School of Informatics, Xiamen University, Xiamen 361005, China; h.esmaiel@aswu.edu.eg; 2 Electrical Engineering Department, Faculty of Engineering, Aswan University, Aswan 81542, Egypt; 3 School of Informatics, Xiamen University, Xiamen 316005, China; zeyadqasem@stu.xmu.edu.cn (Z.A.H.Q.); naveedrehmanjunejo@stu.xmu.edu.cn (N.U.R.J.); 4 Department of Information and Communication Engineering, School of Electrical and Electronic Engineering, Tianjin University of Technology, Tianjin 300383, China; great_seal@163.com

**Keywords:** IoUTs, spatial modulation, underwater communication, unequal error protection, SPIHT coder, image processing

## Abstract

A spatial modulation (SM) scheme has been developed as a hopeful candidate for spectral and energy-efficient wireless communication systems, as it provides a great judgment for the system performance, data transmission rate, receiver complexity, and energy/spectrum efficiency. In SM, the data is conveyed by both habitual M-ary signal constellations and the transmit antennas indices. Therefore, the system data rate improvement due to the side information bits transmitted, encapsulated in indices of the transmit antennas, improves the SM transmission efficiency compared to the different MIMO players. The information bits transmitted over the antenna index and data symbol constellation using M-ary signal performance have different levels of bit error rate (BER) performance. This paper proposes unequal error protection (UEP) scheme for image transmission over the Internet of Underwater Things (IoUTs) using SM. The Set Partitioning in Hierarchical Trees (SPIHT) coders encode the underwater image and classify the encoded bits in two categories: critical and uncritical bits. The critical bits are transmitted over the SM index bits and have a low BER while the uncritical bits are transmitted over high order M-ary signal constellation to resolve the underwater acoustic channel bandwidth limitation problem. The proposed SM-UEP technique has been developed carefully with enough justification and evaluation over the measured underwater acoustic channel and the simulated channel. The simulation results show that the proposed SM-UEP can increase the average peak signal-to-noise ratio (PSNR) of the reconstructed received image considerably, and significantly.

## 1. Introduction

Approximately 71% of the Earth’s surface is water-covered; this water is usually split between the oceans and small-scale seas, but the oceans have ~96.5% of Earth’s water. Ocean warmth command environment and wind originals that change life on earth. Freshwater in lakes and rivers comprises less than one percent of the Earth’s surface water. Countless efforts have been made by the researchers and scientists to discover this undiscovered massive amount of oceans water, but unfortunately the much of the ocean remains undiscovered. The developments in the last several decades in hardware and communication techniques have guided current advances in underwater activities, such as environmental monitoring, moneymaking or research exploration, and harbor protection. The employment of the Internet of Underwater Things (IoUTs) is an optimal system for these tasks. The IoUTs are described as a global network of intelligent, interconnected underwater things that allow controlling of large unexplored water areas [1]. The edge devices collected information is transferred to the sink node, this gathered information can be analyzed and serviced by using ground support units.

Generally, underwater communication is still a big challenge due to the oceanic environment physical characteristics. There are four communication technologies used as a physical layer for underwater communication: optical, electromagnetic, magnetic induction, and acoustic waves. Until now, the acoustic waves is the most used one in long distance underwater communication [2,3]. However, the acoustic bandwidth is limited, and it consumes high power transmission and IoUTs nodes are battery-based with no capability for recharging. Therefore, multimedia data transmitting, such as image and video, is a big challenge especially for real-time IoUTs applications. The next generation of the underwater acoustic communication techniques should be focused on improving transmission data rate to support real-time underwater multimedia applications. However, a high-speed wireless transmission over underwater channel is more complicated compared to the radio links [4].

Underwater multimedia applications can be improved via two research directions: First, by using high-compression encoder techniques, and the second direction is by using effective communication schemes that deal with underwater acoustic channel bandwidth limitations. In line with the first research direction, set partitioning in hierarchical trees (SPIHT) image coding has been proposed and introduced in [4]. The SPIHT algorithm is providing the best peak signal-to-noise ratio (PSNR) condition of the obtained decoded image for addressing compression ratio. The SPIHT coder based on wavelet algorithm is the most employed algorithm in image compression as well as being an essential type of compression for all subsequent algorithms. Spatial modulation technologies have been proposed to deal with the second research direction [3,5]. There are some other research articles, where researchers tried to tackle with improving the underwater multimedia application using combined ideas of two research directions: unequal error protection (UEP) techniques via hierarchical quadrature amplitude modulation (HQAM) [4,6]. In the UEP, the robustness of the transmitted image quality can be significantly increased. The key of UEP approaches can be classified into three main types: (1) joint source-channel coding, by providing the optimal source/channel coding rate; (2) rate allocation, by supplying and optimizing fixed channel rate over source packets; and (3) utilizing modulation systems given different protection levels to different conveyed bits. In line with UEP utilizing modulation systems, this paper proposes a new UEP scheme based on spatial modulation for image transmission over IoUTs to combine high compression SPIHT encoder technique with highly efficient transmission spatial modulation (SM) scheme.

SM [7] is a recently developed transmission technique for wireless communication systems, which uses multiple-input multiple-output (MIMO) antennas. The main idea of SM is conveying additional information in the index of the transmitted antennas: increasing the number of transmit antennas increases the spectral efficiency, and transmitting additional bits without activating all transmit antennas and consume transmitted power. As only one antenna is activated in the SM transmission, SM is a highly energy-efficient modulation technique candidate for the next 5G mobile network [8] and also for IoUTs [9]. There are two information carrying items in SM: (1) constellation signal and (2) index of broadcast antennas with various fault amount. As the SM has two information axes, and each axis has a different level of bit error rate (BER), broadcasting encoded image bits with different level of protection can be applicable. This paper proposes a modified set partitioning in hierarchical trees (M-SPIHT) image coder [6] to generate two different stream of bits based on their significance including critical bits and uncritical bits. The critical bits will be transmitted over the low BER spatial modulation index bits and the uncritical bits will be transmitted via spatial modulation data bits carried using high modulation order scheme to solve the underwater acoustic limited channel bandwidth problem. In this paper, numerical analysis has been used to show the differences in the level of BER between the SM carrying information units. Simulation results have been used to show the error bit sensitivities of the SPIHT encoded bitstream and performance improvement in the reconstructed received underwater image.

In particular, to improve the underwater multimedia transmission over the IoUTs, we have focused on the following aspects in this paper.

1. Encoded image bits classification using M-SPIHT encoder to classify the encoded bits into two categories: critical and uncritical bit streams.
2. Based on the M-SPIHT encoder classification, the UEP using the spatial modulation is proposed to improve the quality of the reconstructed image transmitted over the underwater acoustic channel.
3. A mathematical framework for assessing the ABER performance of the SM information carrying units is laid out thoroughly to prove the capability of UEP using SM scheme.
4. The proposed UEP-SM scheme carefully evaluated the reconstructed image improvement with the PSNR by simulation experiment on the simulated channel and measured underwater acoustic channel.

The rest of the paper is structured as follows. Section 2 represents the SPIHT encoded image classification. Section 3 explains the proposed UEP technique using spatial modulation. The performance analysis and simulation results are discussed in Section 4, and finally the paper is concluded in Section 5.

Notations: Column vectors (matrices) are denoted by boldface lower (upper) case letter; superscripts T, *, and H stand for transpose, conjugate, and conjugate transpose, respectively.

## 2. Encoded Image Classification

Hierarchical SPIHT algorithm is working based on splitting the wavelet coefficients and dependent on a significance-based classification function. These SPIHT wavelet coefficients can be categorized based on the sensitivity of encoded bitstream for error and how is it affect the decoded image. This categorization, based on bit significance, can be written as a classification function as in [10]:

(1) $$S_{n}\left(\Gamma \right)=\left\{\begin{array}{c} 1,\;\mathrm{if}\;\underset{\left(i,j\right)\in \Gamma }{\underbrace{\max}}\left\{\left|Y\left(i,j\right)\right|\geq 2^{n}\right\}\\ 0,\;\mathrm{otherwise}\; \end{array},\right.$$

where $Y\left(i,j\right)$ represents the wavelet coefficient at the pixel $\left(i,j\right)$ for n bit plane. There are two loops in SPIHT algorithm: the sorting loop and the refinement loop. These two sorting loops have three groups of bits [11]: initial indicator of least insignificant pixels, the indicator of least significant pixels, and the indicator of least insignificant sets. Quantization procedure ranks the wavelet coefficients throughout these various groups, utilizing a specific quantization level as in the conventional SPIHT coder. The sorting order will be delivered on split four lists instead of three: roll of the least insignificant sets (LIS), the role of the least insignificant pixels (LIP), the role of the least significant pixels (LSP), and the role of the refinement path. The LSP and LIP consist of the joints and show the individual pixels while the LIS describes the descendant joints. The bits number can express the most significant coefficients as follows,

(2) $$N=log_{2}\left(max_{\left\{i,j\right\}}\left\{\left|Y\left(i,j\right)\right|\right\}\right)$$

The registered LIP of pixels can be done dependent on the importance level by applying Equation (1). The resultant $S_{n}\left(\Gamma \right)$ is then promoted to the coded bits. All those important pixels will be transferred to the LSP organization with their sign bit forwarded to the product stream. Therefore, organizations in LIS will be examined and forwarded to LSP in the state of doing extraordinary, or more into LIP. For refinement loops, the n -th observation significant bit of the coefficients in the LSP is utilized. By iteratively acting for each n that is decreased by one, the required rate can be reached till every LSP node is considered. A various kind of bits for a varied rate of vulnerability to the errors can be achieved using the SPIHT sorting algorithm. Any tiny mistake of significant coded bits is more severe, and on the other hand, errors of other bits have low sensitivity. The bits of the coded SPIHT algorithm is divided into four separate classes; significance bit, sign bits, set bits to determine if setting significantly, and refinement bits. These four classes can be divided based on bits sensitivity into two groups: critical bits (the significance and sign bits) and uncritical bits (set and refinement bits). The critical bits are bits make the synchronization failure within the decoder and encoder, and the reconstructed method can be a mess in case of an error in these critical bits. On the other hand, the uncritical bits make smaller error level, and the result of this error is a coefficient. These critical bits performed and produced through the inspection of the position bits and the refinement bits.

## 3. UEP Using Spatial Modulation

In this section, the specifics of transmitted underwater data image using UEP SM scheme will be presented starting with a brief introduction about SM scheme. Then, the general numerical framing for the average bit error rate (ABER) of signal waveforms for each SM information carrying unit is calculated. At the end of this section, the detailed UEP-SM scheme is explained.

### 3.1. Spatial Modulation

The SM has been introduced based on the constellation set of data symbols sent via the active antenna out of sending antennas [12]. The index of the antenna in a transmission is used to send extra data bits. Therefore, high data rate can be attained in SM compared to space shift keying modulation (SSK) or the conventional multiple-input-multiple-output (MIMO). In SM, the upcoming bitstream sent at a time immediate is divided within two different groups of bits. One of these groups is applied to modulate a signal constellation symbol from a signal constellation diagram of an optional M -ary quadrature amplitude modulation (M -QAM), or any further signal constellation design. These modulated-based bits embed $log_{2}\left(M\right)$ bits, which are called data bits. The other set of bits embeds, $log_{2}\left(Nt\right)$ bits, are characterized as spatial bits, and Nt is the number of broadcasting antennas used at the transmitter side. The spatial bits are applied to choose the antenna subset, which will be used to transmit the constellation symbol. The possible data rate achieved using SM, $R_{SM}$, can be represented as [12]

(3) $$R_{SM}=log_{2}\left(M\right)+log_{2}\left(Nt\right)$$

The SM has a wireless link with  Nt antenna transmission and Nr antenna receiver and SM map constellation vector are $x={\left[x_{1}\;x_{2}\ldots \ldots \;x_{Nt}\right]}^{T}$ assuming unity constraint power (i.e., $E_{X}\left[X^{H}X\right]=1$). SM activate only one antenna, in this case just one of $x_{a}$ of constellation vector x is nonzero to evade power transmission loss. The received signal of SM can be expressed as

(4) $$r=\sqrt{\rho }HX+v$$

where ρ is the average signal to noise ratio (SNR), H is the uncorrelated underwater acoustic channel impulse response, and v is the additive white Gaussian noise with independent and identically distributed (iid) entries according to $CN\left(0,\;1\right)$. During SM time slot, the active constellation vector can be express as

(5) $$x_{b,q}≜{\left[0\;0\;\ldots x_{b,q}\ldots \;0\;0\right]}^{T}$$

where b is the active antenna index and $x_{b,q}$ is the q -th constellation symbol transmitted over the b antenna. The received signal r in the case of $x_{q}$ is transmitted from the b-th antenna and can be written as

(6) $$r=\sqrt{\rho }h_{b}x_{q}+v$$

where $h_{b}$ is the b-th column of the underwater acoustic channel H. With constant modulation signaling assumption, the received signal detection can be obtained as

(7) $$\hat{b}=\arg_{b}\max\left|\frac{h_{b}^{H}r}{{\left|h_{b}\right|}_{F}}\right|$$

(8) q^=argqmax Re hb^xqHr

where $\hat{b}$ and $\hat{q}$ is the estimated antenna and constellation symbol, respectively, and ${\left|h_{b}\right|}_{F}$ represents the Frobenius norm. The error in the estimated antenna is based on the underwater acoustic channel and the number of the antennas transmission, whereas the error of data symbols is based on the underwater acoustic channel and the constellation modulation order.

### 3.2. ABER of SM Information Carrying Units

In this subsection, the ABER performance of SM has been analyzed to show the deference in BER between the two carrying information units: (1) the symbols that were carried via the antenna index and (2) the symbols that were carried via the signal modulation scheme. This difference in ABER is that the two terms make UEP applicable using SM.

#### 3.2.1. ABER of the Bits Carried by the Antenna Index

The occurred error probability on the estimated bits transmitted via the antenna index is derived as follows [13],

(9) $$BER_{AI}\;\leq \;E_{b}\left[\underset{\overset{ˇ}{b}}{\sum }N\left(b\to \tilde{b}\right)\;PEP\left(x_{b}\to x_{\tilde{b}}\right)\right]=\;\frac{1}{2^{R}}\;\overset{2^{R}}{\underset{v=1}{\sum }}\overset{2^{R}}{\underset{k=1}{\sum }}\frac{N_{e}\left(b\to \tilde{b}\right)\;PEP\left(x_{b}\to x_{\tilde{b}}\right)}{R}$$

where $E_{b}$ is the expected transmit antennas, data transmitted rate in case of b antenna index activated is R in Equation (9). The number of bit errors between antenna index b and estimated antenna index $\tilde{b}$ is indicates by $N_{e}\left(b\to \tilde{b}\right)$. $PEP\left(x_{b}\to x_{\tilde{b}}\right)$ is the pairwise error probability $\left(PEP\right)$ which can be written as

(10) $$PEP\left(x_{b}\to x_{\tilde{b}}\right)=\;E_{H}\left\{PEP\left(x_{b}\to x_{\tilde{b}}/H\right)\right\}$$

where $E_{H}\left\{.\right\}$ is the second-order statistic of pairwise error probability over the underwater acoustic channel, and $PEP\left(x_{b}\to x_{\tilde{b}}|H\right)$ is the conditional PEP, and can be written as

(11) $$PEP\left(x_{b}\to x_{\tilde{b}}|H\right)=PEP\left(∥r-\;\sqrt{\rho }hb_{x}{∥}_{F}>∥r-\;\sqrt{\rho }h_{\tilde{b}}x{∥}_{F}\right)$$

Using r as in (4), the conditional PEP can be written as

(12) $$PEP\left(x_{b}\to x_{\tilde{b}}|H\right)=\;PEP\left(∥v{∥}_{F}>∥v+\;\sqrt{\rho }h_{b}x-\;\sqrt{\rho }h_{\tilde{b}}x{∥}_{F}\;\right)=\;Q$$

where,

(13) $$Q\left(z\right)=\;\frac{1}{\pi }\overset{\frac{\pi }{2}}{\underset{0}{\int }}e^{\frac{z^{2}}{sin^{2}\theta \;}}d\theta$$

and (11) can be rewritten as

(14) $$PEP\left(x_{b}\to x_{\tilde{b}}|H\right)=Q\left(\sqrt{\frac{\rho }{2}∥h_{b}x-\;h_{\tilde{b}}x{∥}_{F}^{2}}\right)=Q\left(\sqrt{g}\right)=\;\overset{N_{r}}{\underset{t=1}{\sum }}c_{n}^{2}$$

where $c_{n}\sim \;N\left(0,\;\sigma_{c}^{2}\right)$ with $\sigma_{c}^{2}=\;\frac{\rho }{2}{\left|x\right|}^{2}$ and $g=\frac{\rho }{2}∥h_{b}x-\;h_{\tilde{b}}x{∥}_{F}^{2}$ defined in ([14]; Equation (15)) and it is assumed as a chi-squared random variable (RV) with $2N_{r}$ degree of freedom (DOF), and its probability density function (PDF) can be given as in [13]:

(15) $$F_{g}\left(\psi \right)=\;\frac{\psi^{N_{r}-1}\;e^{\frac{-\psi }{2}}}{2^{N_{r}}\;\Gamma \left(N_{r}\right)}$$

$\Gamma \left(.\right)$ is gamma function. Representing the pairwise error probability in terms of chi-squared RV, g can be written as [15]

(16) $$PEP\left(x_{b}\to x_{\tilde{b}}\right)=\;\overset{\infty }{\underset{0}{\int }}Q\left(\sqrt{\psi }\right)\frac{\psi^{N_{r}-1}\;e^{\frac{-\psi }{2}}}{2^{N_{r}}\;\Gamma \left(N_{r}\right)}\;d\psi =\frac{1}{\pi }\overset{\infty }{\underset{0}{\int }}\int_{0}^{\left(\pi /2\right)}Q\left(\sqrt{\;\psi }\right)\frac{\psi^{N_{r}-1}\;e^{\frac{-\psi }{2}}}{2^{N_{r}}\;\Gamma \left(N_{r}\right)}\;d\psi .\;$$

Then the closed form of Equation (8) can be written as follows [16,17],

(17) $$PEP\left(x_{b}\to x_{\tilde{b}}\right)=\;{\left[\frac{1-\;\mu_{a}}{2}\right]}^{N_{r}}\overset{N_{r}-1}{\underset{w=0}{\sum }}\left(\begin{array}{c} N_{r}-1+w\\ w \end{array}\right){\left(\frac{1-\;\mu_{a}}{2}\right)}^{w}$$

and $\mu_{a}=\left(1-\;\sqrt{\frac{\sigma_{a}^{2}}{1+\sigma_{a}^{2}}}\right)$. Based on [5] the PEP at high SNR can be simplified as

(18) $$PEP\left(x_{b}\to x_{\tilde{b}}\right)≅\;{\left(\frac{1}{4\mu_{a}}\right)}^{N_{r}}\left(\begin{array}{c} 2N_{r}-1\\ N_{r} \end{array}\right)$$

Finally, based on (9)–(18), the bit error rate (BER) of bits carried by the antenna index can be expressed as

(19) $$BER_{AI}\leq \;\frac{1}{2^{R}}\;\overset{2^{R}}{\underset{v=1}{\sum }}\overset{2^{R}}{\underset{k=1}{\sum }}\frac{N_{e}\left(b\to \tilde{b}\right)\;{\left(\frac{1}{4\mu_{a}}\right)}^{N_{r}}\left(\begin{array}{c} 2N_{r}-1\\ N_{r} \end{array}\right)}{R}$$

#### 3.2.2. ABER of the Bits Carried by Constellation Diagram

In conventional SM, the ML decoder is used to detect the information bits carried by the constellation modulation diagram. The estimated transmitted symbol using ML decoder $x_{\hat{q}}$ represented in Equation (8) can, alternatively, expressed as follows [18],

(20) $$x_{\hat{q}}\;=\;arg\;\underset{x_{q}}{\underbrace{min}}\;\left[\sqrt{\rho }∥u_{b,q}{∥}_{F}^{2}\;-\;2Re\left\{r_{\tilde{b}}^{H}u_{b,q}\right\}\right],$$

where $x_{\hat{q}}$ is referred to the q ^th^ symbol of the constellation diagram, r is the vector of received signal, $u_{b,q}=\;h_{b}x_{q}$, $q\;\in \left[1:M\right]$, and M is the modulation order. The error probability constellation diagram over underwater acoustic channel by using the maximum-receive ratio combining (MRRC) can be expressed as [19]

(21) $$SER=\;\frac{\ell }{ℵ}\left\{\frac{1}{2}{\left(\frac{2}{\varkappa \rho +2}\right)}^{N_{r}}-\;\frac{\ell }{2}{\left(\frac{1}{\varkappa \rho +1}\right)}^{N_{r}}+\;\left(1-\ell \right)\overset{ℵ-1}{\underset{w=1}{\sum }}{\left(\frac{B_{w}}{\varkappa \rho +\;B_{w}}\right)}^{N_{r}}+\;\overset{2ℵ-1}{\underset{w=ℵ}{\sum }}{\left(\frac{B_{w}}{\varkappa \rho +\;B_{w}}\right)}^{N_{r}}\right\}.\;$$

ℵ is the number of Monte Carlo, $\ell =\;\left(1-\frac{1}{\sqrt{M}}\right),\;\varkappa =\frac{3}{M-1},\;m=\;log_{2}\left(M\right),\;B_{w}=2sin^{2}\theta_{w},\;\theta_{w}=w\pi /4n$ and the bit error rate of bits carried by constellation diagram $BER_{C}$ can be expressed as follows,

(22) $$BER_{C}\approx \;\frac{SER}{m}$$

The total ABER of the SM can be calculated as

(23) $$ABER_{SM}=\frac{\left(\left(log_{2}\left(Nt\right)\right)\times BER_{AI}\right)+\left(\left(log_{2}\left(M\right)\right)\times BER_{C}\right)}{R_{SM}}$$

### 3.3. UEP Using Spatial Modulation

**Figure 1:**
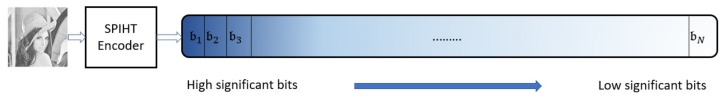
SPIHT progressive source encoder.

**Figure 2:**
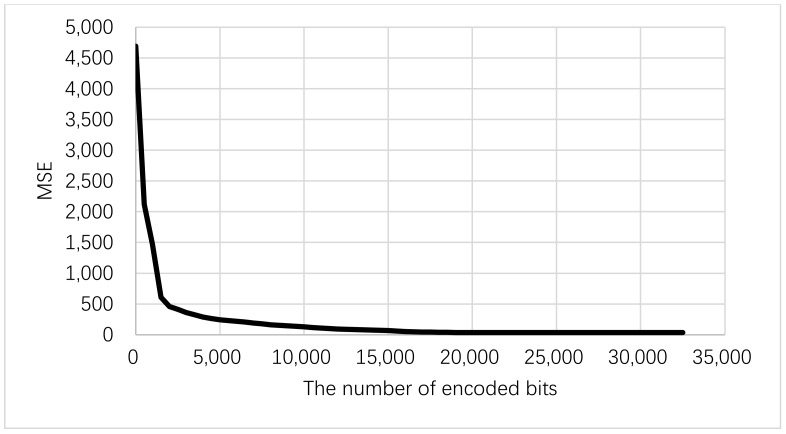
Error bit sensitivities within the SPIHT encoded bitstream.

The source coder used for underwater image transmission should be a low computational complexity encoder to manage the low power feeding of IoUTs nodes, and it must also attain a sufficient compression ratio performance to deal with underwater acoustic limited channel bandwidth. Therefore, many researchers provide a SPIHT coder as a best candidate for underwater image transmission [20,21]. This subsection discusses the UEP technique using a SPIHT coder and SM scheme. The SPIHT coder outputs contain bitstream with unequal importance rank and significance as depicted in Figure 1 [20]. Each encoded bit is more significant than the next one. In UEP using rate allocation, the bits with higher importance level are encoded with high redundancy channel coder [4,6]. These significant bits are shown in Figure 2, to show the effect and contribution of each encoded bit of SPIHT encoder bitstream on PSNR of the reconstructed image. The bit error sensitivity (BES) of SPIHT encoder bitstream is achieved by primary encoding the original transmitted image using the SPIHT encoder and only one bit in the encoded image is corrupted, starting from the first bit to the last one. Each time a bit is corrupted, the encoded image is decoded and the resultant mean square error (MSE) is attained to evaluate its effect on the PSNR of reconstructed image. The corrupted bit is fixed before scheduled on to the next bit.

Figure 2 shows the order of significance from the most significant types of bits to the least significant one of gray (256 × 256) Lena image encoded using SPIHT encoder to generate the source bitstream with a 0.5 bit rate comparison (0.5 bpp). The PSNR value of the reconstructed image is calculated using the following equation [6],

(24) $$PSNR=10log_{10}\left(\frac{M_{G}}{MSE}\right)$$

(25) $$MSE=\frac{1}{A\times B}\underset{Tx\left(𝒶,𝒷\right)}{\sum }\underset{Rx\left(𝒶,𝒷\right)\;}{\sum }{\left[Tx\left(𝒶,𝒷\right)-Rx\left(𝒶,𝒷\right)\right]}^{2}$$

where $Tx\left(𝒶,𝒷\right)$ and $Rx\left(𝒶,𝒷\right)$ are the gray values of pixels in the transmitted and received images, respectively. A and B are image width and height, respectively, and $M_{G}$ is the maximal gray value of the encoded image.

**Figure 3:**
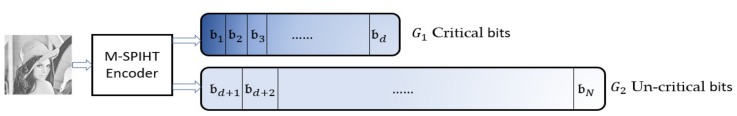
M- SPIHT progressive source encoder.

**Figure 4:**
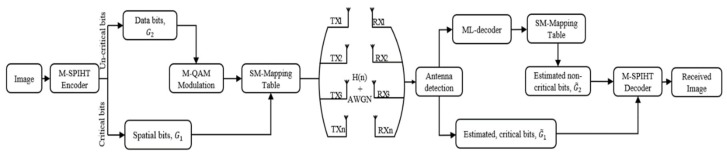
System model.

As shown in Figure 2, the encoded SPIHT bitstream has unequal BES, therefore we can divide the output bitstream to two groups of bits based on its effect on the quality of reconstructed image. This group of bits can be critical and noncritical bits as shown in Figure 3. The N SPIHT encoded bits required to spread over the underwater channel will be divided into two groups, $G_{1}$ and $G_{2}$, and each group has different length of bits. As shown in Figure 4, the high significant critical group of bits $G_{1}$ will be transmitted over the low ABER antennas indices carrying information unit and its length should not exceed the length of bits carried via the transmit antennas indices, and it can be written as

(26) $$G_{1}=\left\{b_{1},b_{2},b_{3},\ldots \ldots \ldots \ldots ,b_{d}\right\},\;\mathrm{and}\;d=\left[\frac{\log_{2}\left(Nt\right)}{R_{SM}}\right]*N$$

The low significant encoded group of bits $G_{2}$, which will be transmitted over SM signal constellations carrying information units can be given by

(27) $$G_{2}=\left\{b_{d+1},b_{d+2},\ldots \ldots \ldots \ldots ,b_{N}\right\}$$

**Figure 5:**
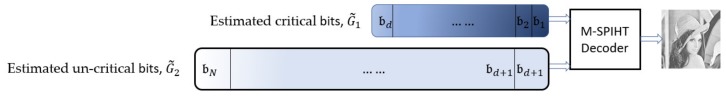
M- SPIHT progressive source decoder.

As shown in UEP-SM scheme, Figure 4, the two groups of M-SPIHT bits, critical bits $G_{1}$, and uncritical bits $G_{2}$ are transmitted as follows; the critical group of bits $G_{1}$ are carried via the antenna index information, and the uncritical group of bits $G_{2}$ are carried via the constellation diagram information carrying units. $G_{1}$ bits are used to choose the index of the activated transmit antennas and $G_{2}$ bits are carried via the constellation diagram of M-QAM modulation then transmitted over the active antenna. At the receiver side, the $G_{1}$ received bits are recovered based on the SM antenna detection mapping table and the modulated uncritical received bits $G_{2}$ are recovered by using ML decoder and M-QAM demodulation. Then the two groups (critical and uncritical) bits are used to decode the M-SPIHT decoder to recover the transmitted image at the receiver side. M-SPIHT decoder will set the received bits as $\tilde{G}=\left[{\tilde{G}}_{1},{\tilde{G}}_{2}\right]$ to reconstruct the received image as shown in Figure 5.

UEP-SM is dividing the encoded SPIHT bitstream into two group of bits and transmitting each group of bits over one of SM transmitting antenna carrying information unit. The MSE of reconstructed image in the UEP-SM will be reduced, as the high significant bits with high MSE contribution in the reconstructed image is transmitted over a low BER SM transmitting carrying information unit and transmitting the low significant bits with low MSE contribution in reconstructed image transmitting over the high BER SM carrying information unit. In other words, despite the ABER of the SM and UEP-SM being equal, the effect of this error is unequal on the MSE of the reconstructed image. The MSE of the reconstructed transmitted image using conventional SM and proposed UEP-SM can be written as

(28) $$MSE\;\left(SM\right)=ABER_{SM}*\overset{N}{\underset{n=1}{\sum }}MSE\left(n\right)$$

(29) $$MSE\;\left(UEP-SM\right)=BER_{AI}\overset{d}{\underset{n=1}{\sum }}MSE\left(n\right)+BER_{C}\overset{N}{\underset{n=d+1}{\sum }}MSE\left(n\right)$$

The proposed UEP-SM can be applied for any of space modulation techniques (SMTs) not only SM. SM-UEP can also be used in the concept of control/user (C/U) plane splitting in the underwater wireless communication sensor network [22,23] or next 5G mobile network [24], by transmitting the control signal over the low ABER index symbols and transmitting the user data plane over high ABER data symbols.

## 4. Simulation Results

**Figure 6:**
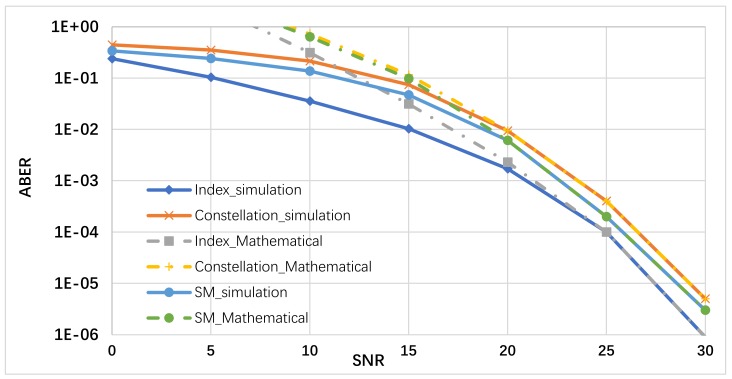
ABER performance comparison between the constellation transmitted symbol, antennas index, and total ABER of SM at 9 bpcu spectral efficiency.

The ABER performance between the received bits of index antennas and the constellated symbols and the ABER performance of index/data SM received bits have been evaluated numerically using Monte Carlo simulations as shown in Figure 6. The ABER performance has been evaluated by using Equations (19), (22), and (23) to get the upper bound of the antennas index, the constellation symbol, and total SM ABER. Then, these asymptotic ABER analytical formulations are confirmed by using Monte Carlo simulations. The simulation results are gotten for 10^6^ symbols conveyed at each SNR for 10^6^ iterations over the simulated channel and the measured underwater acoustic channel collected from sea experiment, ASCOT01 conducted off the coast of New England in June 2001 as reported in [25] and used in [26]. The ABER performance for SM (index/data) and the unseparated SM are shown in Figure 6, using SM technique with 9-bit per channel use (9-bpcu) using SM with Nt=4 and 128-QAM modulation. As shown in Figure 6, the analytical outcomes display a high constancy with the simulation results gotten for SM scheme for the realistic SNR values. However, at the low SNR values, the analytical results disclose a small discrepancy to the simulation results gotten for the ABER of the constellation and index antennas mathematically. In other words, the upper bound is slack to the simulation results at low SNR values, but it stiffens to the simulation results at the realistic SNR values. This agrees with other obtained results with similar bound as in [5,27,28,29,30]. As shown in Figure 6, the difference between the ABER of transmitted symbols and antennas index in the SM is about 8.5 times. Although the unseparated SM ABER has similar performance using SM (data). Therefore, the separation of transmission of critical and noncritical image bitstream over antennas index and data symbols are highly motivated for a different level of data protection in SM, respectively. To evaluate the proposed UEP-SM, the SPIHT coder is applied for (256 × 256) “Lena” and dolphin swimming underwater image [6], using wavelet coefficients to generate the source bitstream with a 0.5 bpp and the SPIHT encoder provide N=32,754 bits. In conventional SM, these bits are transmitted directly via SM based on the SM lock-up table; however, in the proposed UEP-SM, the SPIHT encoded bits are divided based on their significance into two groups: $G_{1}=\left[1:7278\right]$ bits and $G_{2}=\left[7279:32,754\right]$ bits. The incoming M-SPIHT $G_{1}$ bits are transferred via index antenna selector, and $G_{2}$ is transferred via the modulation constellation sector. Using the progressive transmission image ability of the SPIHT coder, the sets of distortion in the received image are obtained from the same received bit stream. The M-SPIHT decoder reads the first bytes of the received bit stream $\tilde{G}=\left[G_{1},G_{2}\right]$ and calculates the inverse sub-band. By calculating the inverse sub-band transformation and then comparing the recovered image with the original, the distortion can be measured by using PSNR as follows,

(30) $$PSNR=10{\;\log}_{10}\left(\frac{255^{2}}{MSE}\right)\;\mathrm{dB}$$

where MSE is the square of mean error between the original and reconstructed image and calculated in Equation (25). The SM-UEP has been compared in terms of the PSNR for the received image with the conventional SM under assumption of using the same Nt and M-QAM at same SNR of underwater channel. Reconstructed “Lena” and dolphin swimming underwater image transmitted via UEP-SM over underwater acoustic channel are shown in Table 1 and Table 2, respectively.

As shown in Table 1, the SM-UEP is improving the PSNR of received “Lena” image by 4.92 dB and 7.1 dB at channel SNR = 10 dB and SNR = 20 dB, respectively. For dolphin swimming underwater image [6], the proposed SM-UEP also improves the PSNR of received dolphin swimming underwater image by 3 dB and 4.1 dB gain at underwater channel has SNR = 10 dB and SNR = 20 dB, respectively.

In Table 1 and Table 2, the performance has been evaluated over the measured underwater acoustic channel, whereas the performance of Table 3 has been evaluated over the simulated channel where the channel tabs are generated based on multipath fading channel. Table 3 shows the proposed SM-UEP in comparison with the conventional SM scheme. The proposed SM-UEP in comparison with the conventional SM improves the PSNR of received “Lena” image by 4.63 dB at low SNR (SNR = 10 dB) and the performances are nearly same at high SNR (SNR = 20 dB). The simulation results clearly show that the proposed SM-UEP is suitable for the underwater image transmission, where it highly overcome the conventional SM especially in highly noisily channel.

## 5. Conclusions

In this paper, UEP is proposed using SM technique for effective image transmission over the underwater acoustic channel. The proposed UEP scheme is settled for SPIHT coded images. The SPIHT critical bits of the compressed underwater image are transmitted over transmit antennas indices with low ABER, while the uncritical bits of the compressed image are transmitted over the habitual M-ary signal constellation of the SM. The proposed UEP scheme has been evaluated over a measured underwater acoustic channel in a sea experimentation state in the past as well as the simulated channel. For measured underwater acoustic channel, the simulation results show that the whole distortion can be efficiently decreased in the reconstructed image and the PSNR can be enhanced by 3.96 dB and 5.6 dB gain at channel SNR = 10 dB and SNR = 20 dB, respectively. In the simulated channel, the PSNR of reconstructed image has been improved by 4.63 dB gain with SNR = 10 dB and provides the same performance as the conventional SM scheme at SNR = 20 dB. The proposed SM-UEP can also be utilized to any other scalable source coder or the concept of C/U plane splitting used in the modern wireless mobile network for different level of data protection.

## Figures and Tables

**Table 1:** Decoded “Lena” image over an underwater acoustic channel with conventional SM and proposed SM-UEP (© 2013 IEEE).

	SNR = 10 dB	SNR = 20 dB
Conventional SM	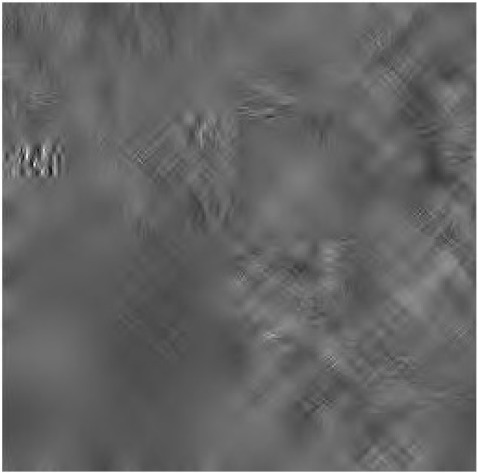	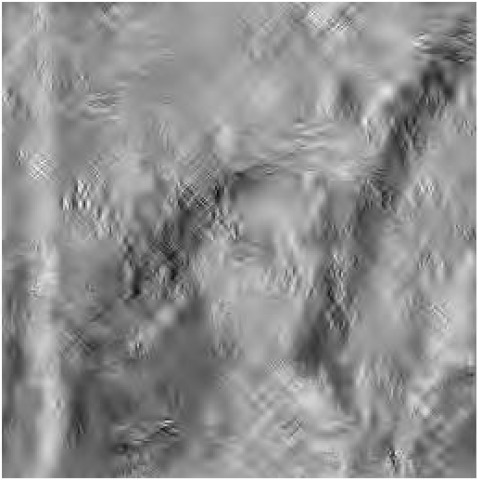
	PSNR = 13.1 dB	PSNR = 20.4 dB
Proposed SM-UEP	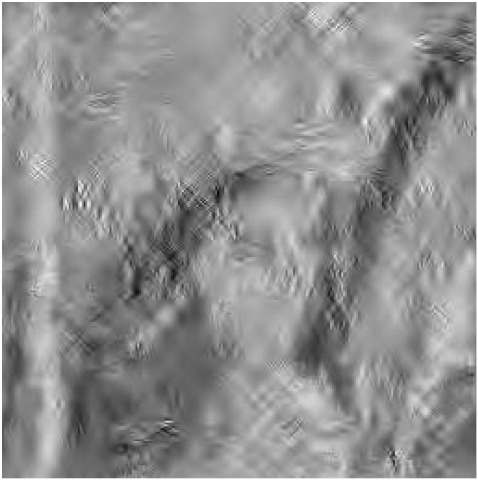	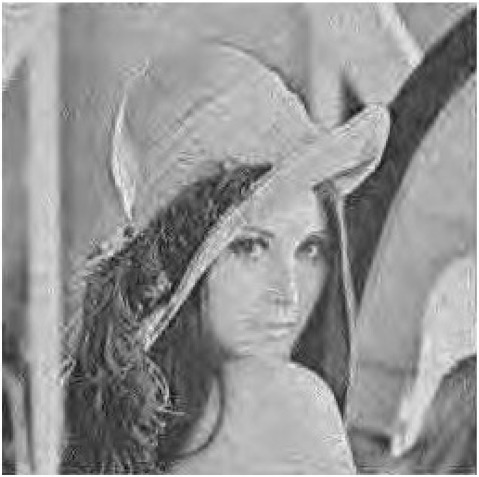
	PSNR = 18.02 dB	PSNR = 27.5 dB

**Table 2:** Decoded dolphin swimming underwater image [6] over an underwater acoustic channel with conventional SM and proposed SM-UEP (© 2013 IEEE).

	SNR = 10 dB	SNR = 20 dB
Conventional SM	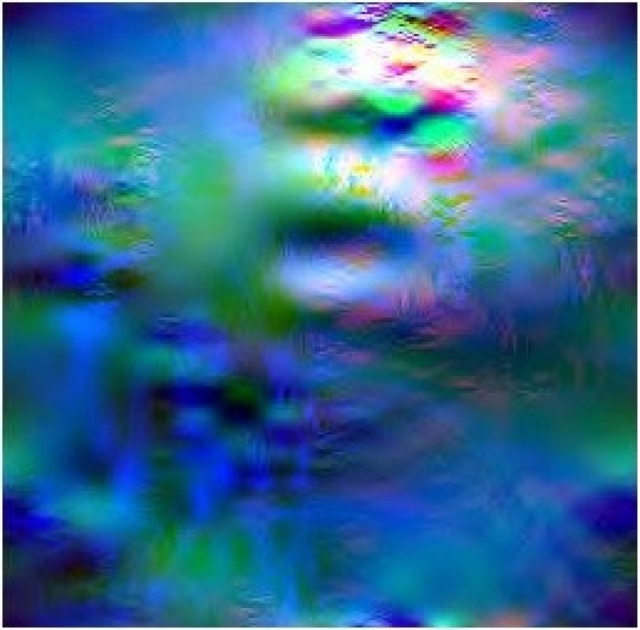	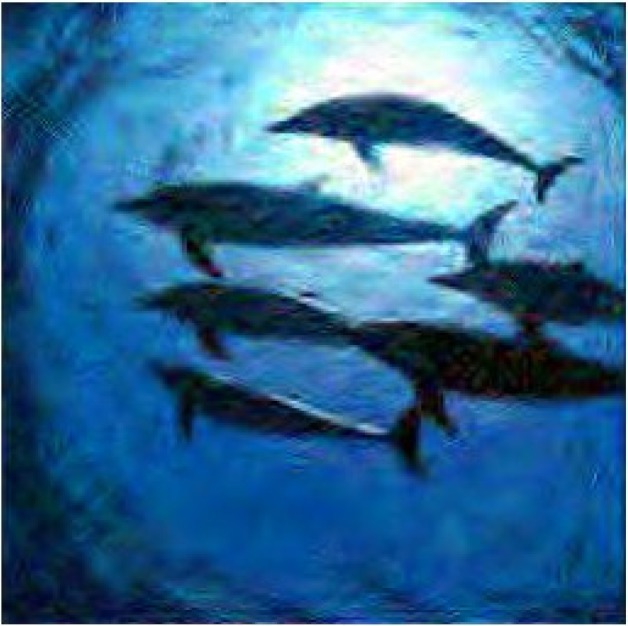
	PSNR = 11.8 dB	PSNR = 23.3 dB
Proposed SM-UEP	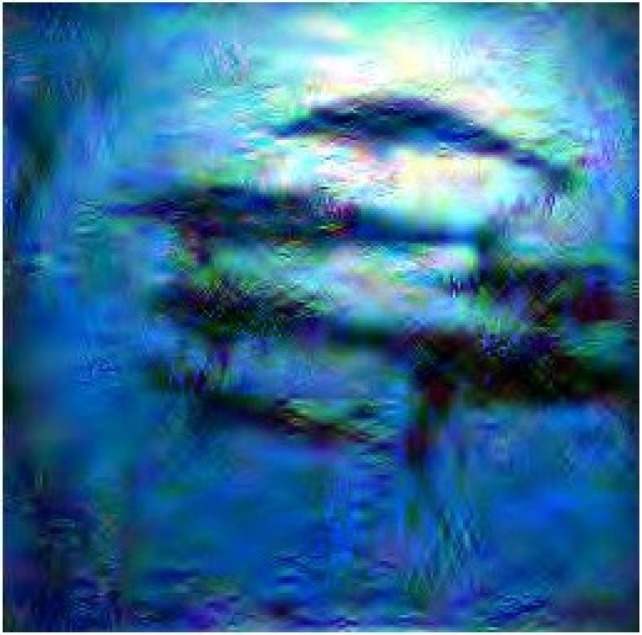	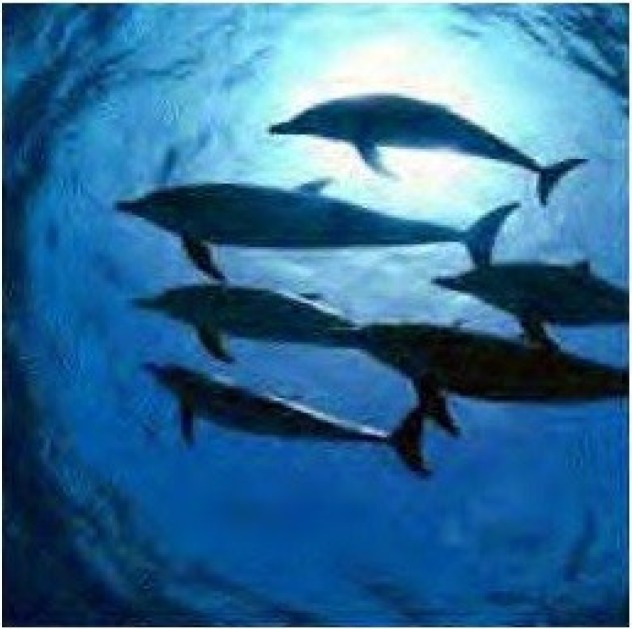
	PSNR = 15.8 dB	PSNR = 27.4 dB

**Table 3:** Decoded “Lena” image over the simulated channel with conventional SM and proposed SM-UEP.

	SNR = 10 dB	SNR = 20 dB
Conventional SM	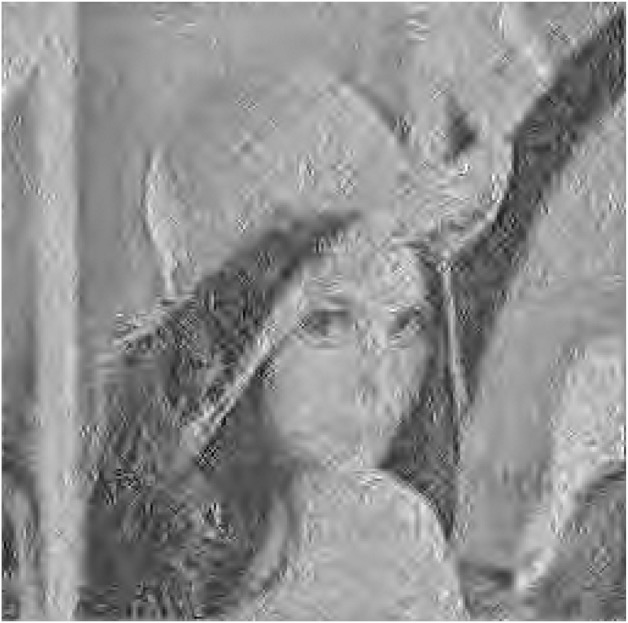	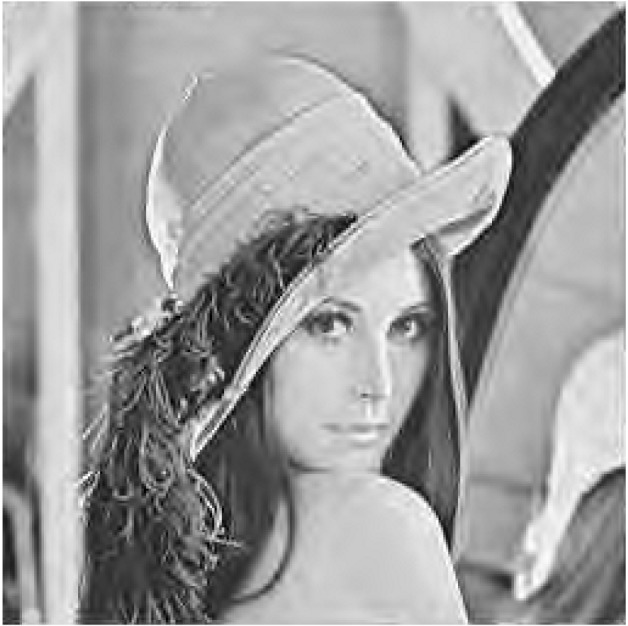
	PSNR = 21.08 dB	PSNR = 32.52 dB
Proposed SM-UEP	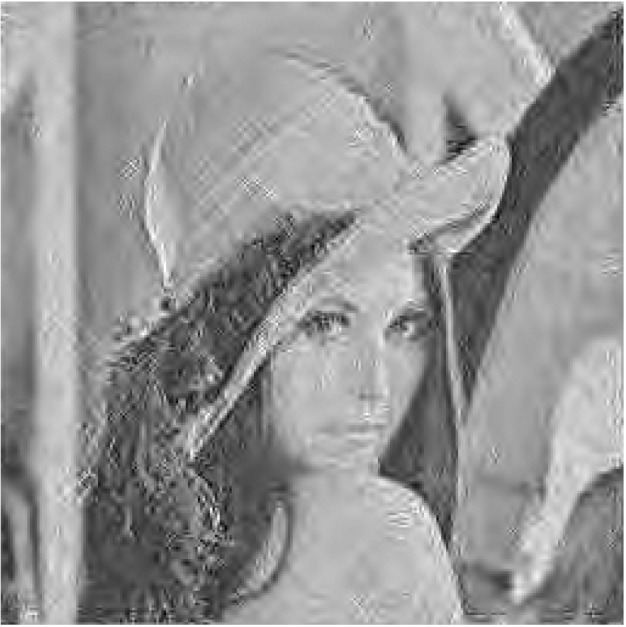	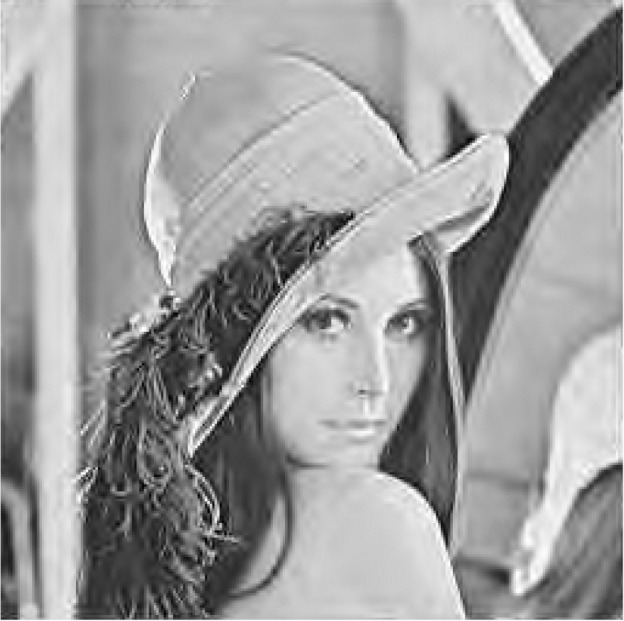
	PSNR = 25.71 dB	PSNR = 32.54 dB
